# Serotonergic gene-to-gene interaction is associated with mood and GABA concentrations but not with pain-related cerebral processing in fibromyalgia subjects and healthy controls

**DOI:** 10.1186/s13041-021-00789-4

**Published:** 2021-05-12

**Authors:** Isabel Ellerbrock, Angelica Sandström, Jeanette Tour, Silvia Fanton, Diana Kadetoff, Martin Schalling, Karin B. Jensen, Rouslan Sitnikov, Eva Kosek

**Affiliations:** 1 Department of Clinical Neuroscience, Karolinska Insitutet, Nobels väg 9, 17177 Stockholm, Sweden; 2 Department of Neuroradiology, Karolinska University Hospital, Stockholm, Sweden; 3 Department of Oncology, Blekinge Hospital, Karlskrona, Sweden; 4 Stockholm Spine Center, Löwenströmska Hospital, Upplands Väsby, Sweden; 5 Department of Molecular Medicine and Surgery, Karolinska Institutet, Stockholm, Sweden; 6 Center for Molecular Medicine, Karolinska University Hospital, Stockholm, Sweden; 7 MRI Research Center, Karolinska University Hospital, Stockholm, Sweden; 8 Department of Surgical Sciences, Uppsala University, Uppsala, Sweden

**Keywords:** Serotonin, GABA, Genetic polymorphisms, 5‐HT1_A_, Serotonin transporter, Fibromyalgia, Mood

## Abstract

The neurotransmitter serotonin, involved in the regulation of pain and emotion, is critically regulated by the 5‐HT_1A_ autoreceptor and the serotonin transporter (5-HTT). Polymorphisms of these genes affect mood and endogenous pain modulation, both demonstrated to be altered in fibromyalgia subjects (FMS). Here, we tested the effects of genetic variants of the 5‐HT_1A_ receptor (CC/G-carriers) and 5-HTT (high/intermediate/low expression) on mood, pain sensitivity, cerebral processing of evoked pain (functional MRI) and concentrations of GABA and glutamate (MR spectroscopy) in rostral anterior cingulate cortex (rACC) and thalamus in FMS and healthy controls (HC). Interactions between serotonin-relevant genes were found in affective characteristics, with genetically inferred high serotonergic signalling (5-HT_1A_ CC/5-HTT_high_ genotypes) being more favourable across groups. Additionally, 5‐HT_1A_ CC homozygotes displayed higher pain thresholds than G-carriers in HC but not in FMS. Cerebral processing of evoked pressure pain differed between groups in thalamus with HC showing more deactivation than FMS, but was not influenced by serotonin-relevant genotypes. In thalamus, we observed a 5‐HT_1A_-by-5-HTT and group-by-5-HTT interaction in GABA concentrations, with the 5-HTT high expressing genotype differing between groups and 5‐HT_1A_ genotypes. No significant effects were seen for glutamate or in rACC. To our knowledge, this is the first report of this serotonergic gene-to-gene interaction associated with mood, both among FMS (depression) and across groups (anxiety). Additionally, our findings provide evidence of an association between the serotonergic system and thalamic GABA concentrations, with individuals possessing genetically inferred high serotonergic signalling exhibiting the highest GABA concentrations, possibly enhancing GABAergic inhibitory effects via 5-HT.

**Supplementary Information:**

The online version contains supplementary material available at 10.1186/s13041-021-00789-4.

## Introduction

Within the central nervous system, serotonin (5-hydroxtryptamine, 5-HT) is synthesized in the brainstem raphe nuclei, with serotonergic projections ascending throughout the brain. The 5-HT metabolism is critically influenced by the main inhibitory serotonergic 5‐HT_1A_ receptors and the serotonin transporter (5-HTT). Presynaptic 5‐HT_1A_ autoreceptors on serotonergic neurons control 5-HT release in terminal areas and decrease neuron firing, resulting in inhibited serotonergic signalling [1, 2]. Activation of postsynaptic 5‐HT_1A_ receptors, largely found in corticolimbic areas [3, 4], produces physiological responses determined by the target cell, e.g. antidepressant and antinociceptive effects. Additionally, 5-HTT regulates serotonergic signalling via 5-HT reuptake from the synaptic cleft into the pre-synaptic neuron, therefore controlling availability and duration of 5-HT effects [5, 6]. Given that serotonergic projections modulate a multitude of behavior, mechanisms influencing 5‐HT signalling are widely implicated in pain, mood and emotion [7–11].

Polymorphisms of serotonergic genes have been shown to affect mood [8, 12, 13] and pain modulation [14–16]. Specifically, an abundance of inhibitory 5‐HT_1A_ autoreceptors has been associated with the G-allele of a single nucleotide polymorphism in the 5‐HT_1A_ receptor gene (*rs6295*) [17–19], linked to lower serotonergic signalling. Moreover, the low expression 5-HTT genotype has been linked to reduced binding in pre- and postsynaptic 5‐HT_1A_ receptors [20–22]. Based on our previous study showing similar effects of the 5-HTT_low_ and the 5‐HT_1A_ */G genotypes on pain regulation in fibromyalgia subjects (FMS) and healthy controls (HC) [23], we would expect pain-relevant genotype interactions. Similar observations have been made in response to selective serotonin reuptake inhibitors (SSRIs) in depression [24–26], providing evidence for a serotonergic gene–gene interaction.

Dysfunctional pain inhibition has been demonstrated in FMS [23, 27, 28], with lower pain-related activation in rostral anterior cingulate cortex (rACC) and thalamus, and reduced functional connectivity within the descending pain system [29, 30]. Reduced 5-HT in cerebrospinal fluid [31] and increased frequency of 5-HTT_low_ in FMS [32] point to a dysregulated 5-HT system. 5-HT is involved in controlling nociceptive transmission, where it can exert inhibitory or facilitatory effects [10, 33], and in common FM comorbidities, e.g. depression [9, 34]. Glutamate and ɣ-aminobutyric acid (GABA), the key excitatory and inhibitory neurotransmitters in the brain, are crucially involved in nociceptive transmission [35] and have been found to be altered in some brain regions in FMS [36, 37]. A recent study suggests widespread alterations in FMS in the GABAergic system [38]. While 5-HT as well as glutamate and GABA have been associated with mood [9, 39, 40] and modulating pain [11, 35, 41], they are also intricately linked in their actions [42]. 5-HT modulates glutamate- and GABA-mediated effects in the spinal dorsal horn and may also elicit inhibitory effects on GABAergic neurons via 5‐HT_1A_ receptors, e.g. in thalamic interneurons [43]. Specifically, higher serotonergic activity increases GABA release, thereby enhancing GABAergic inhibition and modulating sensory signals in the thalamus [43].

Here, we investigated the phenotypic influence of 5‐HT_1A_ and 5-HTT polymorphisms in FMS and HC on (i) mood and pain-relevant characteristics, (ii) cerebral processing of evoked pressure pain in rACC and thalamus using functional magnetic resonance imaging (fMRI), and (iii) glutamate and GABA in rACC and thalamus using magnetic resonance spectroscopy (MRS). We hypothesized that the 5‐HT_1A_ CC and 5-HTT_high_ genotypes, associated with more efficient serotonergic signalling, elicit a favourable effect regarding mood, pain sensitivity and influence cerebral pain processing. The analyses concerning glutamate and GABA are considered exploratory.

## Materials and methods

### Sample

The sample (n = 127) consisted of 84 FMS (mean 47.2 ± 7.8 years) and 43 HC (mean 48.2 ± 7.6 years), recruited as part of a larger project (see study plan https://osf.io/8zqak) [44–46]. Inclusion/exclusion criteria are described in Additional file 1.

### Procedure

Data were collected over two consecutive days: on day one all participants provided saliva samples for genotyping and filled out questionnaires regarding pain catastrophizing (Pain Catastrophizing Scale, PCS [47]), depression (Beck’s Depression Inventory, BDI [48]; Hospital Anxiety and Depression Scale, HAD-D [49]), anxiety (State-Trait Anxiety Inventory, STAI [50] and Hospital Anxiety and Depression Scale, HAD-A [49]) and reported current pain intensity (Visual analogue scale, VASnow). FMS also completed the Fibromyalgia Impact Questionnaire (FIQ) [51]. Higher scores indicate a higher degree of severity in all questionnaires (see Additional file 1 for details).

Pressure pain thresholds (PPTs) were determined to assess pain sensitivity. The handheld pressure algometer (Somedic Sales AB, Hörby, Sweden) had a round 1cm^2^ rubber probe that was applied perpendicular to the surface. Manual force was applied at a steady rate (approximately 50 kPa/s) and subjects pressed a response-button at the first sensation of pain [52]. PPTs were collected bilaterally across four anatomical sites with one assessment per site: supraspinatus muscle, elbow (lateral epicondyle), gluteus muscle and knee (medial fat pad proximal to the joint line). The average individual PPT across body sites was used in the analyses.

Pressure stimuli in the experimental pain paradigm were applied to participants’ left calf using a cuff (13 × 85 cm) attached to a rapid cuff inflation system (E20/AG101, Hokanson, Bellevue, WA, USA). Stimulus intensity was individually calibrated to match 10 mm (P10) and 50 mm (P50) on a VAS ranging from 0 mm (no pain) to 100 mm (strongest imaginable pain). The procedure is described in Additional file 1 and in [45, 46].

On day two, participants underwent a MRI session, including the pressure pain paradigm during fMRI, in which color cues predicted the following stimulus intensity (described in detail in [45, 46]). In short, a green circle (2 s) was followed by the individually calibrated low intensity pressure stimulus (P10, 5 s) and a red circle (2 s) was followed by the individually calibrated medium intensity stimulus (P50, 5 s). The green and red cues and subsequent pressure stimulations, P10 and P50, respectively, were each presented ten times in a pseudo-randomized manner. Participants were prompted to rate pain intensity on a computerized VAS (8 s) after each stimulus.

### MRI data acquisition

All MRI data were collected on a GE MR750 3 T scanner using an 8-channel head coil. Prior to MRS and functional MRI, high-resolution T1-weighted images were acquired (BRAVO, voxel size 1 × 1 × 1 mm, 176 slices).

GABA and glutamate were measured in vivo using single-voxel proton MRS. The voxel position was verified by three-plane localizer images performed before every scan. Before each data acquisition gradient echo shimming, frequency and water suppression adjustments were performed automatically. The conventional point resolved spectroscopy (PRESS) was used with the following parameters: TR/TE/TE1 = 2000/40/19 ms, spectral bandwidth 5 kHz, 4096 time-domain data points and water suppression by three chemical shift selected suppression (CHESS) pre-pulses was used to ensure comparability with other studies. Six sharp outer volume suppression RF pulses surrounded voxel were applied to enhance the voxel definition. The voxel volume was 5.4 mL for rACC and 12 mL for thalamus. Both voxels were acquired with 128 number of averages and 8-step phase cycle, resulting in an acquisition time of 5 min per voxel.

Functional images comprised 42 axial slices (slice thickness 3 mm, 0.5 mm gap) and were acquired using a T2*-sensitive gradient echo-planar imaging sequence (TR 2 s; TE 30 ms; flip angle 70°; field of view 220 × 220 mm, 72 × 72 mm matrix; 3 × 3 mm in-plane resolution).

### Analysis of MRS data

MRS data was pre-processed in MATLAB (The MathWorks, Natick, MA) and quantified in LCModel (version 6.3-1 K, s-provencher.com). The pre-processing included the S/N2-weighted MRS signal coil combining frequency and phase correction for every trace before final coherent averaging of the elementary MRS traces for each voxel. The LCModel basis set was simulated in MATLAB via quantum mechanical density matrix formalism using the PRESS pulse sequence timing parameters and the chemical shifts and J-coupling constants from [53, 54]. The basis set consisted of the following metabolites: aspartate, glutamate, glutamine, GABA, N-acetyl aspartate, myo- and scyllo-inositol, taurine, ascorbate, glucose, creatine and phosphocreatine, choline and glycero-phosphoryl-choline, N-acetyl aspartate-glutamate, glutathione, alanine, lactate, ethanolamine and phosphorylethanolamine. Calibration of the basis set was performed using a MRS phantom (BRAINO + GABA, GE Healthcare). The MRS data were quantified using the ratio to (i) total creatine (7-mM assumed value, relative) and (ii) total voxel water concentration (absolute). Endogenous water concentration was estimated by using MRS voxel co-registration with segmented structural 3D T1-weighted images (grey matter, white matter, CSF) in native space in FSL (version 5, FMRIB Software Library). The obtained tissue volumes were then masked by the voxel and partial volume estimates for each tissue type that was used to correct the total water concentration.

All analyses were carried out separately for rACC and thalamus using the absolute and relative concentrations of glutamate and GABA. MRS data were acquired of 108 participants in rACC (FMS = 68, HC = 40) and 116 participants in thalamus (FMS = 74, HC = 42).

### Analysis of fMRI data

Processing and analysis of functional data was performed using statistical parametric mapping (SPM12, Wellcome Trust Centre for Neuroimaging) running under MATLAB (version R2015b). Anatomical and functional scans were first reoriented manually to the anterior commissure. Volumes were realigned to the mean volume using a six-parameter affine transformation. Then, the anatomical T1-weighted image was coregistered to the functional images. Functional images were spatially normalized to a standard Montreal Neurological Institute (MNI) template and finally smoothed using an isotropic Gaussian kernel (FWHM, 6 mm). Framewise displacement (FD) was used to assess relative head movement from one frame to another using the sum of the absolute values of the derivatives of the realignment parameters [55]. As a consequence, six participants (four FMS, two HC) were excluded from further analyses due to excessive head motion (FD > 0.5 in > 15% of the images). There were no differences in FD between FM and HC (Wilcoxon rank sum test, Z = 1.58, *p* = 0.1145). Data analysis was performed using the general linear model implemented in SPM12-7219. First level analysis included temporal high-pass filtering (cut-off 128 s) and correction for auto-correlations using first-order autoregressive modelling. The following conditions were modelled on the individual level: pressure stimulations for two intensities (P10/P50), two cue/anticipation phases (red preceding P50/green preceding P10) and the rating period. Six realignment-derived parameters capturing motion were added as regressors of no interest.

Region of interest (ROI) analyses were performed to link evoked pain-related blood-oxygen-level-dependent (BOLD)-response (during P50) to metabolites in brain areas relevant to pain-processing and differences between FMS and HC have been observed [29, 30]. Specifically, raw, unscaled parameter estimates (weighted combinations of beta-values) were extracted from individual (first-level) maps for each subject for the contrast P50 > implicit baseline, i.e. inter-trial and waiting periods, averaged within custom-built masks of right rACC and bilateral thalamus, resulting in one value per person and ROI. Masks were created using MarsBar (http://marsbar.sourceforge.net/) with the aim of matching the single MRS voxel. BOLD-signal was averaged over all voxels in brain masks, allowing individually varying number of voxel contributions per subject. This approach was chosen as the extraction of BOLD-response from a mask, i.e. collapsed across voxels within the region, decreases noise and provides comparability with MRS data.

Functional MRI data were collected for 120 participants, of which 15 data sets were excluded from further analysis due to excessive head motion (n = 6), structural brain anomalies (n = 1) and incomplete data sets due to technical issues and drop-outs (n = 8). The fMRI analyses included data of 105 participants, of which four data sets were missing genotyping information. Final results are presented for n = 101 participants (FMS = 68, HC = 33).

### Genotyping

Saliva samples (Oragene G500) were collected from all participants for the purpose of genotyping, which was performed blind to phenotypic information. Individuals were genotyped regarding the 5‐HT_1A_ gene (*rs6295*) and dichotomized into major allele homozygotes (CC) and minor G-allele carriers (CG and GG, i.e. */G) [15, 23].

5-HTT is coded by the SLC6A4 gene, which contains several functional polymorphisms that alter genetic expression, including *5-HTTLPR* (consists of a long allele, L, and a short allele, S) and *rs25531* (A/G substitution), which have been suggested to efficiently study 5-HTT. Given its proposed efficacy [56] and in line with our previous study [23], we examined *5-HTTLPR/rs25531* jointly, resulting in functional groupings of high (L_A_/L_A_), intermediate (L_A_/L_G_ and L_A_/S_A_) and low (S_A_/S_A_ and S_A_/L_G_) expression of 5-HTT.

TaqMan single nucleotide polymorphism genotyping assays and ABI 7900 HT instrument (Applied Biosystems (ABI), Foster City, CA, USA) was used for 5‐HT_1A_ genotyping. Polymerase chain reactions (PCRs), with a total volume of 5 mL, were performed in 384-well plates containing 2.5 mL Universal Master Mix (UMM) and 5 ng dried-down genomic DNA per well. The PCR amplification protocol included 2 holds, 50 °C for 2 min and denaturation at 95 °C for 10 min, followed by 45 cycles at 92 °C for 15 s and 60 °C for 1 min.

Two fragments, 487 bp (short) and 530 bp (long), were amplified by PCRs for the genotyping of the triallelic 5-HTTLPR. Each PCR reaction contained 50 ng DNA, 0.2 mM deoxynucleotide triphosphate (dNTP), 0.4 mMof primer 17P-3F (59-ggcgttgccgctctgaatgc-39), 0.4 mM primer 17P-3R (59-gagggactgagctggacaaccac-39), 0.05 mL Qiagen HotStar Polymerase, 1 M Q-solution, and finally 1 × buffer. Samples were amplified on Biorad Tetrade (BIORAD, Hercules, CA, USA) with an initial denaturation for 10 min at 95 °C followed by 33 cycles consisting of denaturation for 30 s at 95 °C, annealing for 30 s at 57°Cand elongation for 5 min at 72 °C, and finally followed by another elongation step for 5 min at 72 °C. Eight microliters of the PCR reactions were separated for 2 h at 100 V by gel electrophoresis in TBE buffer on a 2.5% agarose gel containing GelRed and visualized using ultraviolet light. To determine *rs25531*, 10 mL of the PCR product was digested with 0.1 mL MSP1 (New England Biolabs, Ipswich, MA, USA) and 1 mL buffer per sample for 12 h at 37 °C. The MSP1 restriction enzyme breaks the 59-C/CGG9 sequence that gives a fragment of 342 base pairs, one of 127 and finally one of 62 base pairs which constitutes the LA allele, whereas the 298, 127, and 62 base pairs is the SA allele, the 173, 166, 127, and 62 base pairs for the LG allele, and finally the 166, 130, 127, and 62 for the SA allele. Fragments were run on a 4% agarose gel (3% normal agarose and 1% low melting agarose) containing GelRed initially for 15 min at 70 V followed by 2 more hours at 100 V. The gels were then visualized with ultraviolet light.

The saliva sample of one FMS was missing and, therefore, not genotyped for 5‐HT_1A_ and 5-HTT. In addition, the 5-HTT genotype could not be determined in two FMS and three HC, as the PCRs did not produce secure read-outs.

### Statistical analyses

Analyses were performed using R version 4.0.3 [57]. ANOVAs and linear mixed effects models were performed using the package *afex* [58]. Follow-up tests using *emmeans* [59] were adjusted for multiple comparisons using Holm’s method [60]. Reported p-values are two-sided and *p* < 0.05 was considered statistically significant.

### Anxiety and pain sensitivity in all participants

Effects on PPTs as a measure of pain sensitivity, calibrated input pressure, as well as state and trait anxiety (STAI) were analyzed in separate ANOVAs with group (FMS/HC), 5‐HT_1A_ (CC/G-carriers) and 5-HTT (high/intermediate/low expression) as factors.

### Clinically relevant characteristics in FMS

Depression (BDI, HAD-D), current pain (VASnow) and pain catastrophizing (PCS) were first tested for group differences using Wilcoxon test or Welch test. Next, these pain-relevant measures and overall FM impact (FIQ) were tested for effects of 5‐HT_1A_ and 5-HTT in separate ANOVAs in FMS only. This approach was taken as acute pain and depression were exclusion criteria for HC, thus, the data lacked sufficient variability and the focus of this study was serotonin-related genes and their potential interaction. Note that opposed to the STAI, the HAD-A was considered a clinical measure, given its clinical target group.

A linear mixed model was performed to test for differences in calibrated input pressure with fixed effects pressure level (P10/P50), group, 5‐HT_1A_ and 5-HTT, including all two-way interactions and random intercept per subject. Variance of the random effect was estimated using restricted maximum likelihood and Satterthwaite approximation was used for degrees of freedom to obtain p-values.

### fMRI and MRS data

In separate analyses for right rACC and bilateral thalamus, group (two levels), 5‐HT_1A_ (two levels) and 5-HTT (three levels) were tested as predictors with the outcome variables (i) extracted BOLD-signal during evoked pain (P50), (ii) glutamate and (iii) GABA. Each ANOVA tested for interactions between 5‐HT_1A_ and 5-HTT, as well as between group and each gene. Glutamate and GABA analyses were performed twice, for relative and absolute metabolite levels.

### Correlations and mediation analysis

Spearman’s rho was used to correlate BOLD-response during evoked pain and baseline MRS data in rACC and thalamus to test for relationships between glutamate concentrations, GABA concentrations and BOLD-signal, respectively.

Next, we explored whether GABA mediated the group effect on pain-evoked BOLD-signal. The hypothesis is that negative BOLD-signal is driven by decreased neuronal activity linked to inhibitory GABAergic neurotransmission. GABA would be considered a significant mediator if the indirect effect was significant while the previously direct group effect on BOLD-signal became nonsignificant after accounting for GABA.

## Results

Frequencies of 5‐HT_1A_ and 5-HTT genotypes showed no significant relationship (*p* = 0.44, Fisher's exact test), negating potential concerns of confounding effects on the tested phenotypes. Genotype frequencies were comparable between groups (Additional file 2: Table S1).

### Mood, anxiety and pain-relevant characteristics

As expected, significant group differences were observed in BDI, HAD, PCS and VASnow, with FMS showing higher current pain, higher levels of anxiety, depression and pain catastrophizing (Table 1). Additional FMS characteristics are presented in Table 1.

**Table 1:** Participant characteristics and group differences for clinical parameters

	FMS (n = 84)	HC (n = 43)	Group difference	
Age	47.2 (7.8)	48.1 (7.6)	*t*(87) = − 0.63	*p* = 0.53
VASnow	53.3 (22.0)	2.2 (3.2)	*t*(90) = 20.80	*p* < 0.001
PCS	18.3 (10.8)	4.7 (7.0)	W = 3170	*p* < 0.001
HAD-A	7.9 (4.3)	3.1 (2.9)	*t*(116) = 7.47	*p* < 0.001
HAD-D	7.2 (4.0)	1.1 (1.5)	*t*(116) = 12.29	*p* < 0.001
BDI	16 (7.7)	0.5 (1.4)	W = 3544	*p* < 0.001
Tenderpoints	16.4 (1.8)	–	–	–
Pain duration (months)	185.6 (104.2)	–	–	*–*

Reported are mean (standard deviation) and Wilcoxon Rank Sum test or Welch two-sample *t*-test

*FMS*  fibromyalgia subjects, *HC*  healthy controls, *VASnow * current pain on visual analogue scale, *PCS* Pain Catastrophizing Scale, *HAD-A*  Hospital Anxiety and Depression Scale (anxiety subscale), *HAD-D* Hospital Anxiety and Depression Scale (depression subscale), *BDI* Beck’s Depression Inventory

In FMS, there were no effects of 5‐HT_1A_ or 5-HTT in pain catastrophizing (PCS), current pain (VASnow), anxiety (HAD-A), FM impact (FIQ) or pain duration (Table 2).

**Table 2:** ANOVAs for pain-relevant characteristics in fibromyalgia subjects

	Effect	*df*_*Num*_*, df*_*Den*_	*MSE*	*F*	*η*^*2*^_*G*_	*p*
Pain duration						
	5‐HT_1A_	1, 74	10,091	0.41	0.005	0.523
	5-HTT	2, 74	10,091	1.34	0.033	0.268
	5‐HT_1A_*5‐HTT	2, 74	10,091	1.79	0.044	0.174
VASnow						
	5‐HT_1A_	1, 75	477.05	0.364	0.005	0.548
	5-HTT	2, 75	477.05	0.369	0.009	0.693
	5‐HT_1A_*5‐HTT	2, 75	477.05	0.903	0.023	0.410
*PCS*						
	5‐HT_1A_	1, 74	116.77	1.66	0.021	0.201
	5-HTT	2, 74	116.77	1.38	0.034	0.258
	5‐HT_1A_*5‐HTT	2, 74	116.77	1.37	0.034	0.260
HAD-A						
	5‐HT_1A_	1, 75	18.68	1.377	0.0168	0.244
	5-HTT	2, 75	18.68	0.932	0.0228	0.398
	5‐HT_1A_*5‐HTT	2, 75	18.68	1.809	0.0443	0.171
HAD-D						
	**5‐HT**_**1A**_	**1, 75**	**11.90**	**5.05**	**0.045**	**0.028**
	**5-HTT**	**2, 75**	**11.90**	**3.92**	**0.070**	**0.024**
	**5‐HT**_1A_*5‐HTT	**2, 75**	**11.90**	**11.68**	**0.210**	** < 0.001**
BDI						
	5‐HT_1A_	1, 74	49.72	3.59	0.034	0.062
	**5-HTT**	**2, 74**	**49.72**	**5.63**	**0.106**	**0.005**
	**5‐HT**_1A_*5‐HTT	**2, 74**	**49.72**	**8.67**	**0.163**	** < 0.001**
*FIQ*						
	5‐HT_1A_	1, 75	269.59	0.03	0.001	0.870
	5-HTT	2, 75	269.59	1.34	0.032	0.268
	5‐HT_1A_*5‐HTT	2, 75	269.59	2.86	0.069	0.064

*PCS*  Pain Catastrophizing Scale, *VASnow*  current pain on visual analogue scale, *HAD-A*  Hospital Anxiety and Depression Scale (anxiety subscale), *HAD-D* Hospital Anxiety and Depression Scale (depression subscale), *BDI* Beck’s Depression Inventory, *FIQ* Fibromyalgia Impact Questionnaire, *df*_*Num*_  numerator degree of freedom, *df*_*Den*_  denominator degrees of freedom, *MSE*  mean square error, *η*^*2*^_*G*_ generalized eta-squared. Effects significant at *p* < 0.05 are depicted in bold

**Fig. 1:**
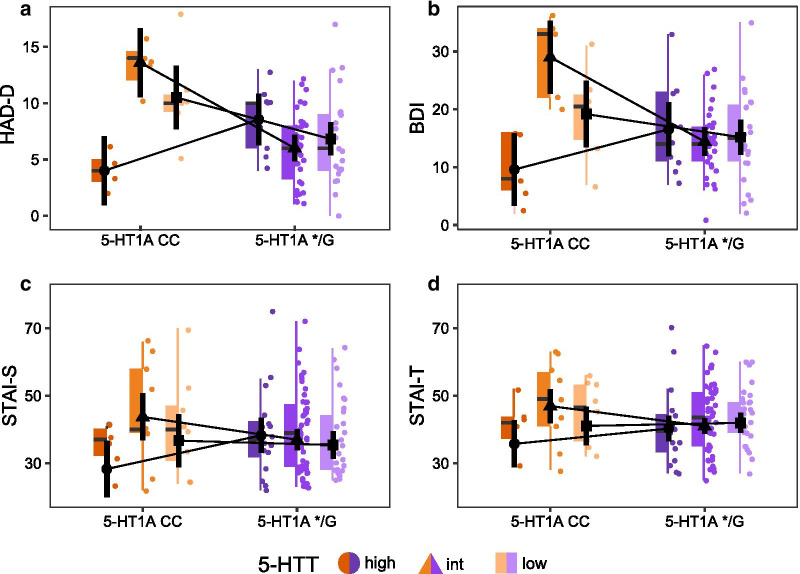
Gene-by-gene interaction in measures of depression in fibromyalgia subjects (FMS) (n = 81) and anxiety in all participants (n = 120). **a** FMS displayed a significant 5‐HT_1A_-by-5-HTT interaction in HAD-D (Hospital Anxiety and Depression Scale, Depression subscale) scores and **b** BDI (Beck’s depression inventory) with 5‐HT_1A_ CC paired with the 5-HTT high expressing genotype resulting in lowest depression values. **c** A similar pattern was observed in STAI (State-trait anxiety inventory) data obtained from all participants (FMS and healthy controls) with both STAI-S (state subscale) and **d** STAI-T (trait subscale) displaying serotonin-relevant gene–gene interactions in addition to significant group differences (see Additional file 3: Fig. S1). Plotted are black circles, triangles and squares representing estimated marginal means with 95% confidence interval combined with colored boxplots, with the line representing the median, the upper and lower box representing the 25th (Q1) and 75th (Q3) percentile, i.e. the interquartile range (IQR), and whiskers represent Q1− and Q3 + 1.5*IQR. Raw data is plotted in the background

Assessing depressive symptoms in FMS using the HAD-D, there was a significant 5‐HT_1A_-by-5-HTT interaction (Table 2), with 5‐HT_1A_ CC showing higher values and therefore more negative characteristics than */G, unless coupled with 5-HTT_high_. Specifically, 5‐HT_1A_ CC paired with 5-HTT_high_ resulted in lowest values, indicative of less depressive symptoms. Follow-up tests revealed significant differences between 5‐HT_1A_ genotypes for all 5-HTT genotypes (5-HTT_high_: *t*(75) = -2.37, *p* = 0.041; 5-HTT_intermediate_: *t*(75) = 4.60, *p* < 0.001; 5-HTT_low_: *t*(75) = 2.32, *p* = 0.041), albeit in different directions (Fig. 1a). The analysis of BDI scores resulted in a similar pattern (Table 2). Follow-up tests on the 5‐HT_1A_ × 5-HTT interaction in BDI scores revealed that 5‐HT_1A_ genotypes differed significantly in 5-HTT_intermediate_ (*t*(74) = 14.58, *p* < 0.001) but not in 5-HTT_high_ (*t*(75) = -1.77, *p* = 0.162) or 5-HTT_low_ (*t*(75) = 1.23, *p* = 0.224) (Fig. 1b).

Interestingly, an interaction between serotonin-relevant genes was also observed in the analysis of anxiety including both FMS and HC. In state anxiety (STAI-S), a significant 5‐HT_1A_-by-5-HTT interaction (F(2,109) = 3.28, *η*^*2*^_*G*_ = 0.042, *p* = 0.041) was found in addition to FMS displaying more anxiety than HC (mean difference FM-HC = 13.7 points) (F(1,109) = 32.16, *η*^*2*^_*G*_ = 0.207, *p* < 0.001) (Additional file 3: Fig. S1). Similar to analyses of depression in FMS, a homozygous 5‐HT_1A_ CC genotype in combination with high expressing 5-HTT showed lower values, i.e. less (current) anxiety, than individuals with low- vs. intermediate expressing 5-HTT genotypes (Fig. 1c). In follow-up analyses, however, the differences between 5‐HT_1A_ in 5-HTT genotypes were not significant after multiple comparison correction. In trait anxiety (STAI-T), a very similar pattern was found (Fig. 1d), but only significant group differences emerged (mean difference FM-HC = 13.9 points) (F(1,109) = 49.29, *η*^*2*^_*G*_ = 0.285, *p* < 0.001), whereas the 5‐HT_1A_-by-5-HTT interaction did not reach significance (F(2,109) = 8.67, *η*^*2*^_*G*_ = 0.163, *p* = 0.069).

### PPT

PPTs, a measure of pain sensitivity, differed between groups, showing FMS (M = 152, SD = 63) were overall more pain sensitive than HC (M = 311, SD = 99) (F(1,110) = 83.83, *η*^*2*^_*G*_ = 0.386, *p* < 0.001). In addition, a significant group-by-5‐HT_1A_ interaction was observed (F(1,110) = 7.87, *η*^*2*^_*G*_ = 0.036, *p* = 0.006). Follow-up tests revealed that 5-HT_1A_ genotypes differed significantly in HC (*t*(110) = 3.16, *p* = 0.004) but not in FMS (*t*(110) = -0.24, *p* = 0.808). Homozygous C-allele carriers in HC (M = 391, SD = 109) displayed significantly higher PPTs than HC G-carriers (M = 297, SD = 92), while there was no significant difference observed between FMS CC (M = 150, SD = 55) and FMS G-carriers (M = 152, SD = 65) (Additional file 4: Fig. S2a).

### Input pressure

There were no differences between 5-HT_1A_ or 5-HTT or interactions with group or pressure level (Additional file 2: Table S2). As we have previously reported [45, 46], FMS displayed higher pain sensitivity than HC, i.e. they required less pressure stimulation to reach 10/100 VAS and 50/100 VAS, respectively. Additionally, a main effect for the pressure level was found. The effects of group and pressure level were qualified by a significant group-by-pressure interaction (Additional file 4: Fig. S2b), indicating a larger difference between groups in P50 (mean difference HC-FM = 96 mmHg) than P10 (mean difference HC-FM = 52 mmHg).

### fMRI

**Fig. 2:**
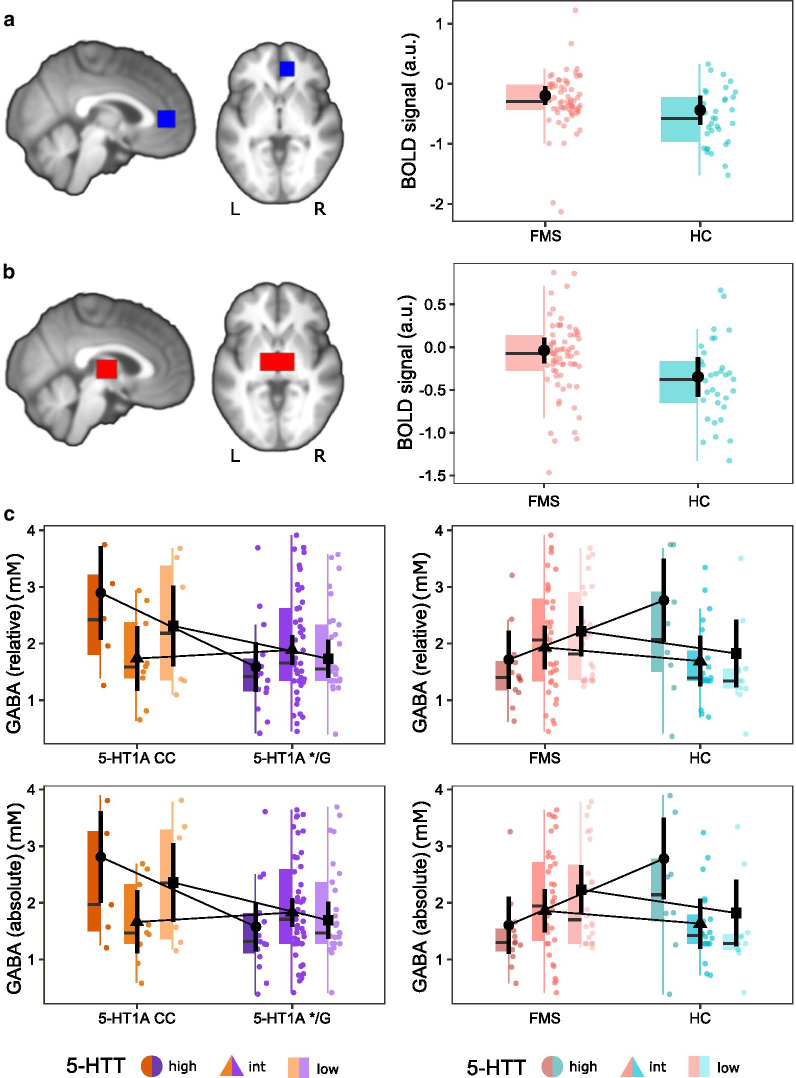
Extracted BOLD-signal and GABA concentration. Fibromyalgia subjects (FMS) (n = 68) showed a similar pattern of lower pain-evoked BOLD-signal as healthy controls (HC) (n = 33) in **a** right rACC and **b** bilateral thalamus. This effect, however, was only significant in thalamus. **c** In thalamic GABA concentrations (FMS = 74, HC = 42), there was a significant 5‐HT_1A_-by-5-HTT interaction, as well as a significant group-by-5-HTT interaction. A comparable pattern was observed in both relative (upper row) and absolute GABA (lower row) concentrations. Plotted are black circles, triangles and squares representing estimated marginal means with the associated black vertical lines being 95% confidence intervals. In the colored boxplots, the horizontal line represents the median, the upper and lower box representing the 25th (Q1) and 75th (Q3) percentile, i.e. the interquartile range (IQR), and whiskers represent Q1− and Q3 + 1.5*IQR. Raw data is plotted in the background. *a.u.* arbitrary units, *mM* millimole, *L* left, *R* right

There were no significance differences between serotonergic genotypes or groups in BOLD-signal during evoked pressure pain in rACC (Table 3, Fig. 2a).

**Table 3:** ANOVAs for extracted BOLD-signal during evoked pain (P50) in rACC and thalamus

	Effect	*df*_*Num*_*, df*_*Den*_	*MSE*	*F*	*η*^*2*^_*G*_	*p*
rACC						
	Group	1, 91	0.22	2.70	0.027	0.104
	5‐HT_1A_	1, 91	0.22	3.21	0.032	0.076
	5-HTT	2, 92	0.22	0.12	0.002	0.887
	Group*5‐HT_1A_	1, 91	0.22	0.16	0.002	0.688
	Group*5‐HTT	2, 92	0.22	1.67	0.033	0.194
	5‐HT_1A_*5‐HTT	2, 92	0.22	0.51	0.010	0.602
Thalamus						
	**Group**	**1, 91**	**0.20**	**4.80**	**0.046**	**0.031**
	5‐HT_1A_	1, 91	0.20	1.00	0.010	0.320
	5-HTT	2, 92	0.20	0.18	0.004	0.834
	Group*5‐HT_1A_	1, 91	0.20	0.30	0.003	0.588
	Group*5‐HTT	2, 92	0.20	2.48	0.048	0.090
	5‐HT_1A_*5‐HTT	2, 92	0.20	0.50	0.010	0.607

*df*_*Num*_  numerator degree of freedom, *df*_*Den*_  denominator degrees of freedom, *MSE* mean square error, *η*^*2*^_*G*_ generalized eta-squared. Effects significant at p < 0.05 are depicted in bold

In thalamus, pain-evoked BOLD-signal compared to implicit baseline differed significantly between groups with HC showing more deactivation than FM (Table 3, Fig. 2b). No effect for 5‐HT_1A_, 5-HTT or any interaction was observed.

### MRS

No significant effects of group, 5‐HT_1A_, 5-HTT or significant interactions were found regarding GABA or glutamate concentrations in rACC (Additional file 2: Table S3). Absolute metabolites based on quantification in relation to tissue water showed similar results.

In thalamus, there was a significant effect of 5‐HT_1A_ with 5‐HT_1A_ CC having higher GABA concentrations (Table 4). This observed main effect was qualified by a statistically significant 5‐HT_1A_-by-5-HTT interaction, with significant 5‐HT_1A_ differences (CC > */G) in 5-HTT_high_ (*t*(101) = 2.80, *p* = 0.018) but not in 5-HTT_intermediate_ (*t*(101) = -0.48, *p* = 0.632) or 5-HTT_low_ (*t*(101) = 1.48, *p* = 0.283) (Fig. 2c). Additionally, a significant group-by-5-HTT interaction was found in GABA, revealing FMS and HC differed in 5-HTT_high_ (*t*(101) = -2.45, *p* = 0.048) (HC > FMS) but not in 5-HTT_intermediate_ (*t*(101) = 0.86, *p* = 0.508) or 5-HTT_low_ (*t*(101) = 1.15, *p* = 0.508) (Fig. 2c). Including a three-way interaction (5‐HT_1A_-by-5-HTT-by-group) did not significantly change the results and was removed from the final analysis. No effect of serotonin-related genotypes or group were found in glutamate. Water-scaled absolute glutamate and GABA concentrations showed similar results (Table 4, Fig. 2c). Raw values of metabolite concentrations are presented in Additional file 2: Table S4.

**Table 4:** ANOVAs for relative and absolute glutamate and GABA concentrations in thalamus

	Effect	*df*_*Num*_*, df*_*Den*_	*MSE*	*F*	*η*^*2*^_*G*_	*p*
Relative glutamate						
	Group	1, 101	1.84	0.55	0.005	0.460
	5‐HT_1A_	1, 101	1.84	1.16	0.011	0.283
	5-HTT	2, 101	1.84	0.20	0.004	0.820
	Group*5‐HT_1A_	1, 101	1.84	0.15	0.001	0.703
	Group*5‐HTT	2, 101	1.84	0.12	0.002	0.890
	5‐HT_1A_*5‐HTT	2, 101	1.84	0.51	0.010	0.603
Relative GABA						
	Group	1, 101	0.73	0.34	0.003	0.563
	**5‐HT**_**1A**_	**1, 101**	**0.73**	**6.05**	**0.047**	**0.016**
	5-HTT	2, 101	0.73	1.19	0.019	0.308
	Group*5‐HT_1A_	1, 101	0.73	1.35	0.011	0.247
	Group*5‐HTT	**2, 101**	**0.73**	**4.67**	**0.073**	**0.011**
	**5‐HT**_1A_*5‐HTT	**2, 101**	**0.73**	**3.60**	**0.056**	**0.031**
Absolute glutamate						
	Group	1, 101	1.65	0.10	< 0.001	0.748
	5‐HT_1A_	1, 101	1.65	0.29	0.003	0.592
	5-HTT	2, 101	1.65	0.40	0.008	0.669
	Group*5‐HT_1A_	1, 101	1.65	0.16	0.001	0.693
	Group*5‐HTT	2, 101	1.65	0.50	0.009	0.611
	5‐HT_1A_*5‐HTT	2, 101	1.65	1.01	0.019	0.367
Absolute GABA						
	Group	1, 101	0.70	0.59	0.005	0.442
	**5‐HT**_**1A**_	**1, 101**	**0.70**	**6.23**	**0.047**	**0.014**
	5-HTT	2, 101	0.70	1.47	0.022	0.236
	Group*5‐HT_1A_	1, 101	0.70	1.47	0.011	0.228
	Group*5‐HTT	**2, 101**	**0.70**	**5.81**	**0.089**	**0.004**
	**5‐HT**_1A_*5‐HTT	**2, 101**	**0.70**	**3.63**	**0.055**	**0.030**

*df*_*Num*_  numerator degree of freedom, *df*_*Den*_  denominator degrees of freedom, *MSE* mean square error, *η*^*2*^_*G*_ generalized eta-squared. Effects significant at *p* < 0.05 are depicted in bold

### Correlations and mediation analysis

There were no correlations between BOLD-signal during evoked pain processing and glutamate or GABA concentrations at baseline in either rACC or thalamus.

We explored whether the BOLD group effect in thalamus during evoked pain was mediated via GABA. Here, group predicted BOLD-response, explaining a significant proportion of variance (R^2^ = 0.05, F(1, 98) = 5.58, *p* = 0.02). However, as group was not a significant predictor of the potential mediator GABA (R^2^ = 0.01, F(1, 98) = 1.27, *p* = 0.26), testing for a mediation effect was unsuitable.

## Discussion

The present multimodal imaging study provides evidence for an association between serotonergic signalling and thalamic GABA concentrations. Regarding thalamic GABA concentrations, a significant effect was found for 5‐HT_1A_, which was qualified by a 5‐HT_1A_-by-5-HTT interaction with higher relative and absolute GABA concentrations in 5‐HT_1A_ CC compared to G-carriers, specifically in the 5-HTT high expressing genotype. Additionally, a group-by-5-HTT interaction revealed higher GABA concentrations in HC than FMS with 5-HTT_high_ but not 5-HTT_intermediate_ or 5-HTT_low_. Our data indicate an association between serotonergic signalling and thalamic GABA levels, with individuals with genetically inferred more pronounced serotonergic signalling (5‐HT_1A_ CC and 5-HTT_high_) presenting with higher GABA concentrations. Furthermore, among 5-HTT_high_ carriers, HC had higher thalamic GABA concentrations than FMS, which is consistent with the lower serotonergic metabolism in FMS [31, 61]. Cerebral processing of evoked pressure pain differed between groups with HC showing more thalamic deactivation than FMS, an effect not mediated by thalamic GABA concentrations, but no significant effects of 5‐HT_1A_ or 5-HTT were observed. No associations were found between serotonergic genotypes and sensitivity to pressure level used to evoke pain in FMS, however, a group-by-5‐HT_1A_ interaction was found in PPTs, with CC homozygotes displaying higher pain thresholds than G-carriers in HC. Finally, significant interactions between the serotonergic genes in mood were found among FMS (depression) and across groups (anxiety). To our knowledge, this is the first evidence of this gene-to-gene interaction indicating a favourable effect on mood of genetically inferred high serotonergic signalling (5‐HT_1A_ CC paired with 5-HTT_high_).

Similar patterns of 5‐HT_1A_-by-5-HTT interactions were observed for measures of mood, with 5-HT_1A_ CC displaying lowest depression scores in combination with 5-HTT_high_ (Fig. 1a, b). This is in line with the assumption that individuals with a more efficient serotonergic system display less depressive symptoms, based on the combined effect of two genotypes producing higher 5-HT signalling. Previous literature points in the same direction: depressed patients with the 5-HT_1A_ CC genotype responded better compared to G-carriers to antidepressants [62, 63] and 5-HTT_low_ individuals with chronic pain displayed higher depression scores than intermediate and high expression genotypes [64]. The apparent vulnerability genotype combination, 5-HT_1A_ */G paired with 5-HTT_low_, resulted in less efficient response to citalopram [24]. Conversely, 5-HT_1A_ CC and 5-HTT_high_ responded better to the SSRI fluoxetine [25]. Some studies have investigated combined effects of 5-HT_1A_ and 5-HTT genotypes, e.g. in major depression, mediating the influence of negative life events [65] and 5-HT_1A_ */G and 5-HTT_low_ showing increased amygdala response to emotional stimuli [66], though results were not entirely consistent [67].

With respect to anxiety in FMS, there were no significant effects of serotonergic genes in HAD-A. This is consistent with the STAI showing mixed results for state and trait subscales, as the HAD covers an intermediate time frame. STAI data of all participants allowed testing for interactions between serotonin-relevant genes and group. Even though groups differed significantly in STAI anxiety scores, no group interaction with 5-HT_1A_ or 5-HTT was observed, indicating a similar effect, regardless of baseline levels. Again, lowest anxiety scores were displayed by 5-HT_1A_ CC in combination with 5-HTT_high_ (Fig. 1c, d). Taking together previous literature and mechanistic function, this corroborates our findings of serotonergic gene-by-gene interactions in measures of mood.

In PPTs we found that 5‐HT_1A_ genotypes differed depending on group, with 5‐HT_1A_ CC in HC, i.e. a higher serotonergic tone, being less pain sensitive than G-carriers. This effect may be absent in FMS as complex modulatory processes attenuate the effect of a favourable genetic makeup. Yet, there was no effect of 5‐HT_1A_ on pressure intensity to achieve 50/100 VAS. This is partially in line with healthy 5‐HT_1A_ G-carriers being less sensitive at thermal pain threshold level but more sensitive at supra-threshold level, indicating a lower serotonergic tone may be associated with a “hypo-to-hyper” response [15].

Similar patterns of HC displaying more BOLD deactivation during evoked pain than FMS were observed in both rACC and thalamus but only significant in thalamus (Fig. 2a, b). BOLD deactivation could implicate a decrease in oxygen consumption and neural activity [68, 69], yet, the neurophysiological interpretation has been a matter of debate [70, 71]. Some studies suggest a negative association between task-related BOLD-response and baseline GABA [72–75], including negative BOLD-response being mediated via GABA [76]. This mechanism may lead to observed group differences, discussed for FM in [77]. However, we found no evidence that the thalamic BOLD-signal group differences during evoked pain were GABA-mediated, adding to the mixed reports on intra-regional connections between baseline GABA concentrations and task-evoked BOLD-response [72, 73, 78, 79]. Functional MRS, however, revealed an increase in glutamate/glutamine and simultaneous decrease in GABA + during painful heat stimulation [80]. Thus, a relationship between pain-relevant metabolites and pain-evoked BOLD-signal may be observed if obtained simultaneously.

Previously, we observed decreased rACC and thalamus activation in FMS compared to HC [29] but a stronger deactivation in HC compared to FMS in the current study. There are methodological differences that may explain the observed mismatch between studies. First, the stimulus differed: whereas a short (2.5 s) sharp pressure stimulus to the thumb nail was used by Jensen (2009), a cuff evoking deep-tissue pain to the leg for 5 s was employed here. This more ecologically valid stimulus may be less efficient in breaking through ongoing processing of perceptually similar FM pain, resulting in subtle differences compared to the baseline. Second, participants included by Jensen (2009) were more impacted by FM and subject to stricter inclusion criteria, as they participated in a pharmacological multicenter study. Current FMS were more heterogeneous, representing realistic variability in FM but making pinpointing underlying causes for discrepancies between studies more challenging. Lastly, earlier studies have found thalamic hypoperfusion in FMS [81, 82], indicating reduced blow flow may contribute to groups differences in BOLD-signal.

We found no significant group differences in GABA or glutamate concentrations in rACC or thalamus, which is consistent with previous findings [36, 83–85]. Whereas no group differences were found per se, groups’ GABA concentrations differed in thalamus between 5‐HTT genotypes, suggesting 5-HTT influences GABA concentration differently, depending on group affiliation. A 5‐HT_1A_-by-5-HTT interaction was also observed in thalamic GABA concentrations, implying that the serotonergic genes elicit a combined effect. As the genotype coding for high 5-HTT expression showed significant differences in GABA concentration between groups and 5‐HT_1A_ genotypes, it is hypothesized that higher serotonergic signalling is more impacted than less pronounced signalling by other mechanisms influencing serotonergic signalling. This would be congruent with an enhanced antidepressant treatment response in 5-HTT_high_ [86–88] and 5‐HT_1A_ CC genotypes [25, 89].

One explanation could speculatively be the involvement of the transcription factor Deaf1 regulating the expression of 5‐HT_1A_ receptors. Contrarily, Deaf1 decreases 5‐HT_1A_ autoreceptor expression in serotonergic raphe cells but upregulates heteroreceptor expression in brain regions receiving serotonergic input [90, 91]. The 5‐HT_1A_ G-allele blocks Deaf1 function, leading G-carriers to have increased 5‐HT_1A_ autoreceptors that reduce 5-HT firing but downregulated postsynaptic 5‐HT_1A_ receptors, resulting in reduced 5-HT neurotransmission. As presynaptic 5‐HT_1A_ receptors may inhibit GABA release [92], carriers of the 5‐HT_1A_ G-allele with reduced presynaptic 5‐HT_1A_ receptors on GABAergic neurons would exhibit higher GABA release into the synaptic cleft. A direct link between GABA-A receptors and the somatic thalamus inhibitory system was observed in a rat model [93], suggesting pain-relevant GABA receptors mediate inhibition via the serotonergic system. Through an increase in GABA via thalamic interneuron dendrites, 5-HT can enhance GABAergic inhibitory effects, thereby modulating sensory processing in the thalamus [43].

Alterations in the transcriptional regulation could also be critical in the underlying mechanisms associated with the 5‐HT_1A_-by-5-HTT interaction. Both polymorphisms are located within the promoter regions of each gene, resulting in altered transcription rates not only when the 5‐HT_1A_ G-allele is present but also in association with the 5-HTTLPR S-allele (5-HTT_low_). Specifically, the S-allele (5- HTT_low_) initiates a reduction in transcription rate of the 5-HTT gene [94]. Taken together, the altered transcription rates affect transporter as well as receptor expression and influence the amount of extracellular serotonin that is available for postsynaptic signalling. Moreover, the 5-HTT low expression genotype has been associated with downregulation of 5‐HT_1A_ heteroreceptors in humans [21]. Lastly, the interplay between serotonergic and GABAergic systems in the CNS is highly complex, with 5-HT intricately exerting modulatory control over glutamate- and GABA-mediated transmission that involves numerous 5-HT receptor subtypes.

### Limitations

Measurement of baseline metabolites in MRS has shown mixed findings in connection with task-related fMRI. Here, functional MRS may be advantageous to understand the dynamics of glutamate/GABA in pain. Additionally, differing genotype frequencies in 5-HTT and 5-HT_1A_ led to differences in subgroup sizes, thus, some analyses may have been underpowered. We emphasize that replication in a larger sample is necessary. Moreover, several neurotransmitters systems are intricately linked [7, 95] but were not accounted for in the current study, given that the sample size is severely limited in brain imaging studies.

## Conclusions

To our knowledge, this is the first report of interactions between these serotonin-relevant genes regarding mood, both in FMS (depression) and across groups (anxiety). Across groups, the 5‐HT1A CC genotype in combination with the 5-HTT high expressing genotype had favourable attributes, possibly associated with producing the highest amount of postsynaptic serotonergic signalling. No significant associations were observed between the serotonergic genotypes and intensity of ongoing pain or pain sensitivity in FMS, while healthy carriers of the 5‐HT1A CC genotype were less pain sensitive. Cerebral pain processing was not associated with serotonergic genotypes, nor with GABA or glutamate concentrations in rACC or thalamus. However, our findings provide evidence of an association between the serotonergic system and GABA levels in the thalamus, with individuals with genetically inferred high serotonergic signalling exhibiting the highest GABA concentrations.

## Supplementary Information

**Additional file 1:** Supplementary materials and methods.

**Additional file 2: Table S1**. Genotype frequencies of the polymorphisms 5‐HT_1A_ (*rs6296*) and the triallelic 5-HTT in fibromyalgia subjects (FMS) and healthy controls (HC). **Table S2.** Linear mixed model for calibrated input pressure to reach 10/100 (P10) and 50/100 VAS (P50). **Table S3.** ANOVAs for relative and absolute glutamate and GABA concentrations in rACC. **Table S4.** Absolute and relative glutamate and GABA concentrations in rACC and thalamus.

**Additional file 3: Fig. S1**. Group differences in anxiety scores in fibromyalgia subjects (FMS) (n = 79) and healthy controls (HC) (n = 40). FMS showed significantly higher anxiety scores measured by the STAI (State-trait anxiety inventory) in both STAI-S (state subscale) and STAI-T (trait subscale). Plotted are black circles representing estimated marginal means with the associated black vertical lines being 95% confidence intervals. In the colored boxplots, the horizontal line represents the median, the upper and lower box representing the 25th (Q1) and 75th (Q3) percentile, i.e. the interquartile range (IQR), and whiskers represent Q1—and Q3 + 1.5*IQR. Raw data is plotted in the background.

**Additional file 4: Fig. S2.** Pain sensitivity measures in fibromyalgia subjects (FMS) (n = 80) and healthy controls (HC) (n = 40). A) In pressure pain thresholds (PPT) using a handheld algometer, a group-by-5‐HT_1A_ interaction was observed, with 5‐HT_1A_ CC in HC showing higher PPTs than G-carriers. This effect was not observed in FMS. B) Input pressure delivered via a rapid cuff inflation system necessary to achieve 10/100 on a visual analogue scale (VAS), P10, and 50/100 VAS, P50, differed between groups depending on pressure level. Plotted are black circles representing estimated marginal means with the associated black vertical lines being 95% confidence intervals. In the colored boxplots, the horizontal line represents the median, the upper and lower box representing the 25^th^ (Q1) and 75^th^ (Q3) percentile, i.e. the interquartile range (IQR), and whiskers represent Q1—and Q3 + 1.5*IQR. Raw data is plotted in the background. kPa = kilopascal, mmHg = millimeter of mercury

## Data Availability

The datasets generated and analysed during the current study are available on reasonable request.
