# Supplementary material for: Serotonergic gene-to-gene interaction is associated with mood and GABA concentrations but not with pain-related cerebral processing in fibromyalgia subjects and healthy controls
Source: Mol Brain. 2021 May 12;14:81. doi: 10.1186/s13041-021-00789-4 (PMC8117625; doi:10.1186/s13041-021-00789-4)
Supplement: Supplementary file 1 — Additional file 1: Supplementary materials and methods. [file 13041_2021_789_MOESM1_ESM.docx]

**Additional File 1: Supplementary Materials and Methods**

*Participants*

Interested individuals underwent systematic screening by a specialist in rehabilitation medicine and pain relief (Dr. Kadetoff), which included assurance that ACR-1990 and ACR-2011 [1,2] classification criteria for fibromyalgia (FM) were met. Included FM subjects (FMS) were right-handed females of working age (20-60 years). Individuals were excluded if any of the following exclusion criteria were present: other dominant pain conditions than FM, painful osteoarthritis, rheumatic or autoimmune diseases, other severe somatic diseases (neurological, cardiovascular, cancer, diabetes mellitus etc.), hypertension (>160/90 mmHg), previous brain or heart surgery, psychiatric disorders including ongoing treatment for depression or anxiety, substance abuse, pregnancy, magnetic implants, self-reported claustrophobia, obesity (BMI>35), smoking (>5 cigarettes/day), inability to speak and understand Swedish, medication with antidepressants or anticonvulsants, inability to refrain from analgesics, NSAID or hypnotics prior to study participation (48h before the first visit, and 72h before the second visit, i.e. MRI). HC were right-handed females, age-balanced to FMS, free from chronic pain conditions and without regular medications with NSAIDs, analgesics or sleep medication in addition to the exclusion criteria for FMS. Participants were recruited through newspaper advertisement and received remuneration for study.

*Questionnaires*

The Pain Catastrophizing Scale (PCS) [3] measures pain catastrophizing tendencies on a 13-item scale, with higher scores suggest higher catastrophizing about pain. Beck’s Depression Inventory (BDI) [4] is a 21-item test that assesses depression with higher scores indicating more depressive severity. The State-Trait Anxiety Inventory (STAI) [5] is divided into a State- and Trait-subscale, each consisting of 20 items assessing the current anxiety (STAI-S) or general anxiety (STAI-T), with a score ranging from 20-80 with higher scores indicative of higher levels of anxiety. The Hospital Anxiety and Depression Scale (HAD) targets clinical populations and [6] consists of 14 items with two subscales, composed of statements relevant to either generalised anxiety (HAD-A) or depression (HAD-D) and higher values indicative of higher anxiety or depression, respectively. The Fibromyalgia Impact Questionnaire (FIQ) [7] is a questionnaire assessing FM-specific symptoms and disability. It consists of 20 items with a score ranging from 0-100, where a higher value indicates a poorer state of health.

*Calibration procedure*

Participants were presented with a series of stimulations (5 s) in increasing intensity steps of 25 mmHg. Following each stimulus, subjects rated their perceived pain on a 100 mm (visual analogue scale (VAS). First, cuff pressure pain threshold (first (VAS) rating > 0) and stimulation maximum (first VAS rating > 60) were determined. In two following series, 5 stimuli were presented in a randomized manner to determine the individual representation of P10 (starting from the pain threshold) and P50 (starting from the stimulation maximum). The randomized series to determine P10 used the pain threshold as a starting point and -2 steps and +2 steps of 25mmHg. The randomized series to determine P50 used the stimulation maximum as a starting point and -4 steps of 25mmHg. If the first subjective rating of 10mm VAS was <100 mmHg, steps of 10 mmHg were used for the randomized series determining P10. Next, subjects were trained in front of a computer monitor to associate green circle with their individually calibrated P10 stimulation and a red circle with their individually calibrated P50 stimulation (familiarization phase), presented in a pseudo-randomized order (10 x P10; 10 x P50).

**References**

1. Wolfe F, Clauw DJ, Fitzcharles M-A, Goldenberg DL, Häuser W, Katz RS, et al. Fibromyalgia criteria and severity scales for clinical and epidemiological studies: a modification of the ACR Preliminary Diagnostic Criteria for Fibromyalgia. J Rheumatol. 2011;38:1113–22.

2. Wolfe F, Smythe HA, Yunus MB, Bennett RM, Bombardier C, Goldenberg DL, et al. The american college of rheumatology 1990 criteria for the classification of fibromyalgia. Arthritis Rheum. 1990;33:160–72.

3. Sullivan MJL, Bishop SR, Pivik J. The Pain Catastrophizing Scale: Development and validation. Psychol Assess. 1995;7:524–32.

4. Beck AT, Ward CH, Mendelson M, Mock J, Erbaugh J. An inventory for measuring depression. Arch Gen Psychiatry. 1961;4:561–71.

5. Spielberger C, Gorsuch R, Lushene R, Vagg P, Jacobs G. Manual for the State-Trait Anxiety Inventory. Palo Alto, CA: Consulting Psychologists Press, Inc.; 1983.

6. Zigmond AS, Snaith RP. The Hospital Anxiety and Depression Scale. Acta Psychiatr Scand. 1983;67:361–70.

7. Burckhardt CS, Clark SR, Bennett RM. The fibromyalgia impact questionnaire: development and validation. J Rheumatol. 1991;18:728–33.
