# Supplementary material for: Serotonergic gene-to-gene interaction is associated with mood and GABA concentrations but not with pain-related cerebral processing in fibromyalgia subjects and healthy controls
Source: Mol Brain. 2021 May 12;14:81. doi: 10.1186/s13041-021-00789-4 (PMC8117625; doi:10.1186/s13041-021-00789-4)
Supplement: Supplementary file 2 — Additional file 2: Table S1. Genotype frequencies of the polymorphisms 5‐HT1A (rs6296) and the triallelic 5-HTT in fibromyalgia subjects (FMS) and healthy controls (HC). Table S2. Linear mixed model for calibrated input pressure to reach 10/100 (P10) and 50/100 VAS (P50). Table S3. ANOVAs for relative and absolute glutamate and GABA concentrations in rACC. Table S4. Absolute and relative glutamate and GABA concentrations in rACC and thalamus. [file 13041_2021_789_MOESM2_ESM.docx]

**Additional File 2: Tables**

**Table S1. Genotype frequencies of the polymorphisms 5‐HT_1A_ (*rs6296*) and the triallelic 5-HTT in fibromyalgia subjects (FMS) and healthy controls (HC)**

|  | FMS (n=84) | | HC (n=43) | | Group difference |
| --- | --- | --- | --- | --- | --- |
|  | N | % | N | **%** |  |
| *5‐HT_1A_* |  |  |  |  |  |
| *CC* | 16 | 19.3 | 9 | 20.9 |  |
| *G-carriers* | 67 | 80.7 | 34 | 79.1 |  |
| *Total* | 83 | 98.8* | 43 | 100* | χ^2^=0.02, p=0.95 |
| *5-HTT* |  |  |  |  |  |
| *High* | 14 | 17.3 | 8 | 20 |  |
| *Intermediate* | 39 | 48.1 | 21 | 52.5 |  |
| *Low* | 28 | 34.6 | 11 | 27.5 |  |
| *Total* | 81 | 96.4* | 40 | 93* | χ^2^=0.63, p=0.73 |

Chi-squared tests for group differences in genotype frequencies are reported.

* Percentage of secure read-outs

**Table S2. Linear mixed model for calibrated input pressure to reach 10/100 (P10) and 50/100 VAS (P50)**

| Effect | β | *SE* | *df* | *t* | *p* |
| --- | --- | --- | --- | --- | --- |
| ***Input pressure (P10)*** | **-71.03** | **3.59** | **108** | **-19.80** | **<0.001** |
| ***Group (FMS)*** | **-34.41** | **6.86** | **113** | **-5.02** | **<0.001** |
| *5-HT_1A_* (CC genotype) | -5.87 | 6.72 | 108 | -0.87 | 0.384 |
| *5-HTT high vs. intermediate* | -6.64 | 9.99 | 108 | -0.67 | 0.507 |
| *5-HTT high vs. low* | 6.69 | 8.56 | 108 | 0.78 | 0.436 |
| ***Group*Input pressure (FMS*P10)*** | **11.00** | **2.79** | **113** | **3.94** | **<0.001** |
| *Group*5-HT_1A_ (FMS*********CC)* | 3.90 | 6.54 | 108 | 0.60 | 0.552 |
| *Input pressure*5-HT_1A_ (P10*********CC)* | -0.85 | 3.57 | 113 | -0.25 | 0.801 |
| *Group*5-HTT (FMS*********high)* | -2.93 | 8.57 | 108 | -0.34 | 0.733 |
| *Group*5-HTT (FMS******** *intermediate)* | -1.81 | 6.78 | 108 | -0.27 | 0.789 |
| *Input pressure*5-HTT (P10*********high)* | 2.55 | 4.51 | 113 | 0.57 | 0.573 |
| *Input pressure*5-HTT (P10*********intermediate)* | -1.81 | 3.56 | 113 | -0.51 | 0.612 |
| *5-HT_1A_ *5-HTT (CC*********high)* | -3.69 | 9.58 | 108 | -0.39 | 0.701 |
| *5-HT_1A_ *5-HTT (CC*intermediate)* | 3.81 | 8.31 | 108 | 0.46 | 0.648 |

VAS = visual analogue scale, FMS = fibromyalgia subjects, β = beta estimate, SE = standard error, df = degrees of freedom; The reference factor level is indicated in parentheses. Effects significant at p<0.05 are depicted in bold.

**Table S3**. **ANOVAs for relative and absolute glutamate and GABA concentrations in rACC**

|  | *Effect* | *df_Num_, df_Den_* | *MSE* | *F* | *η^2^_G_* | *p* |
| --- | --- | --- | --- | --- | --- | --- |
| *Relative glutamate* | | | | | | |
|  | Group | 1, 93 | 5.38 | 0.89 | .009 | .349 |
|  | 5‐HT_1A_ | 1, 93 | 5.38 | 0.66 | .007 | .419 |
|  | 5-HTT | 2, 93 | 5.38 | 0.68 | .014 | .508 |
|  | Group*5‐HT_1A_ | 1, 93 | 5.38 | 2.05 | .021 | .155 |
|  | Group*5‐HTT | 2, 93 | 5.38 | 0.47 | .010 | .623 |
|  | 5‐HT_1A_*5‐HTT | 2, 93 | 5.38 | 0.33 | .007 | .719 |
| *Relative GABA* | | | | | | |
|  | Group | 1, 93 | 1.01 | 0.45 | .004 | .502 |
|  | 5‐HT_1A_ | 1, 93 | 1.01 | 0.67 | .006 | .414 |
|  | 5-HTT | 2, 93 | 1.01 | 2.68 | .051 | .074 |
|  | Group*5‐HT_1A_ | 1, 93 | 1.01 | 3.43 | .033 | .067 |
|  | Group*5‐HTT | 2, 93 | 1.01 | 0.97 | .018 | .382 |
|  | 5‐HT_1A_*5‐HTT | 2, 93 | 1.01 | 0.26 | .005 | .770 |
| *Absolute glutamate* | | | | | | |
|  | Group | 1, 93 | 6.28 | 1.42 | .014 | .237 |
|  | 5‐HT_1A_ | 1, 93 | 6.28 | 0.97 | .009 | .353 |
|  | 5-HTT | 2, 93 | 6.28 | 0.63 | .013 | .534 |
|  | Group*5‐HT_1A_ | 1, 93 | 6.28 | 1.90 | .019 | .171 |
|  | Group*5‐HTT | 2, 93 | 6.28 | 0.38 | .008 | .685 |
|  | 5‐HT_1A_*5‐HTT | 2, 93 | 6.28 | 0.45 | .009 | .641 |
| *Absolute GABA* | | | | | | |
|  | Group | 1, 93 | 1.08 | 0.69 | .006 | .409 |
|  | 5‐HT_1A_ | 1, 93 | 1.08 | 0.47 | .004 | .493 |
|  | 5-HTT | 2, 93 | 1.08 | 2.73 | .051 | .071 |
|  | Group*5‐HT_1A_ | 1, 93 | 1.08 | 3.76 | .035 | .056 |
|  | Group*5‐HTT | 2, 93 | 1.08 | 1.26 | .024 | .288 |
|  | 5‐HT_1A_*5‐HTT | 2, 93 | 1.08 | 0.09 | .002 | .914 |

*df_Num_* = numerator degree of freedom, *df_Den_* = denominator degrees of freedom, MSE = mean square error, *η^2^_G_* = generalized eta-squared

**Table S4. Absolute and relative glutamate and GABA concentrations in rACC and thalamus**

|  |  | *Glutamate (mM)* | | *GABA (mM)* | |
| --- | --- | --- | --- | --- | --- |
|  |  | Absolute | Relative | Absolute | Relative |
|  | N | M (SD) | M (SD) | M (SD) | M (SD) |
| *rACC* | | | | | |
| FMS | 68 | 10.85 (2.75) | 10.22 (2.65) | 2.36 (1.11) | 2.22 (1.12) |
| HC | 40 | 10.57 (2.40) | 10.16 (1.78) | 2.38 (0.98) | 2.27 (0.84) |
| 5‐HT_1A_ CC | 21 | 11.33 (2.43) | 10.80 (1.91) | 2.55 (1.06) | 2.45 (1.06) |
| 5‐HT_1A_ G-carriers | 87 | 10.60 (2.65) | 10.05 (2.44) | 2.32 (1.06) | 2.19 (1.01) |
| 5‐HT_1A_ CC FMS | 12 | 12.26 (2.38) | 11.51 (1.82) | 2.97 (1.13) | 2.83 (1.20) |
| 5‐HT_1A_ G-carriers FMS | 56 | 10.54 (2.74) | 9.94 (2.73) | 2.23 (1.07) | 2.09 (1.07) |
| 5‐HT_1A_ CC HC | 8 | 10.00 (2.10) | 9.95 (1.75) | 2.03 (0.69) | 2.00 (0.61) |
| 5‐HT_1A_ G-carriers HC | 32 | 10.72 (2.48) | 10.21 (1.81) | 2.46 (1.03) | 2.33 (0.88) |
| 5-HTT high | 18 | 10.58 (2.57) | 10.11 (1.95) | 2.05 (0.88) | 1.95 (0.88) |
| 5-HTT int | 52 | 10.30 (2.51) | 9.77 (2.37) | 2.32 (1.09) | 2.20 (1.02) |
| 5-HTT low | 33 | 11.29 (2.46) | 10.71 (2.39) | 2.68 (1.07) | 2.54 (1.09) |
| 5-HTT high FMS | 10 | 10.60 (1.87) | 9.92 (1.75) | 2.29 (1.10) | 2.13 (1.11) |
| 5-HTT int FMS | 32 | 10.18 (2.84) | 9.66 (2.78) | 2.23 (1.17) | 2.12 (1.14) |
| 5-HTT low FMS | 24 | 11.55 (2.60) | 10.93 (2.59) | 2.54 (1.07) | 2.40 (1.15) |
| 5-HTT high HC | 8 | 10.56 (3.40) | 10.33 (2.28) | 1.75 (0.41) | 1.71 (0.40) |
| 5-HTT int HC | 20 | 10.48 (1.92) | 9.96 (1.54) | 2.46 (0.95) | 2.32 (0.79) |
| 5-HTT low HC | 9 | 10.11 (1.76) | 10.11 (1.76) | 3.07 (1.00) | 2.91 (0.83) |
| *Thalamus* | | | | | |
| FMS | 74 | 9.30 (1.20) | 9.59 (1.41) | 1.94 (0.89) | 1.99 (0.90) |
| HC | 42 | 8.99 (1.31) | 9.36 (1.14) | 1.67 (0.82) | 1.71 (0.82) |
| 5‐HT_1A_ CC | 22 | 9.26 (1.07) | 9.77 (1.25) | 1.98 (0.98) | 2.03 (0.96) |
| 5‐HT_1A_ G-carriers | 94 | 9.17 (1.29) | 9.44 (1.33) | 1.81 (0.85) | 1.85 (0.87) |
| 5‐HT_1A_ CC FMS | 13 | 9.30 (1.25) | 9.97 (1.33) | 2.02 (0.95) | 2.08 (0.89) |
| 5‐HT_1A_ G-carriers FMS | 61 | 9.30 (1.20) | 9.50 (1.42) | 1.92 (0.89) | 1.97 (0.91) |
| 5‐HT_1A_ CC HC | 8 | 9.15 (0.83) | 9.35 (1.13) | 1.98 (1.14) | 2.01 (1.17) |
| 5‐HT_1A_ G-carriers HC | 34 | 8.96 (1.41) | 9.36 (1.16) | 1.60 (0.72) | 1.63 (0.72) |
| 5-HTT high | 20 | 9.16 (1.04) | 9.47 (0.99) | 1.76 (0.96) | 1.79 (0.94) |
| 5-HTT int | 56 | 9.19 (1.46) | 9.50 (1.44) | 1.87 (0.86) | 1.93 (0.91) |
| 5-HTT low | 35 | 9.25 (0.91) | 9.53 (1.32) | 1.92 (0.37) | 1.94 (0.87) |
| 5-HTT high FMS | 12 | 9.04 (1.13) | 9.51 (0.90) | 1.45 (0.68) | 1.54 (0.70) |
| 5-HTT int FMS | 36 | 9.32 (1.30) | 9.59 (1.50) | 2.02 (0.94) | 2.08 (0.98) |
| 5-HTT low FMS | 24 | 9.39 (1.15) | 9.64 (1.50) | 2.10 (0.89) | 2.11 (0.85) |
| 5-HTT high HC | 8 | 9.34 (0.94) | 9.42 (1.17) | 2.22 (1.16) | 2.17 (1.15) |
| 5-HTT int HC | 20 | 8.94 (1.72) | 9.34 (1.35) | 1.60 (0.65) | 1.66 (0.71) |
| 5-HTT low HC | 11 | 8.93 (0.71) | 9.30 (0.82) | 1.52 (0.80) | 1.56 (0.80) |

FMS = fibromyalgia subjects, HC = healthy controls, mM = millimole, int = intermediate, mean (M), standard deviation (SD)
