# Supplementary figures and images for: Serotonergic gene-to-gene interaction is associated with mood and GABA concentrations but not with pain-related cerebral processing in fibromyalgia subjects and healthy controls
Source: Mol Brain. 2021 May 12;14:81. doi: 10.1186/s13041-021-00789-4 (PMC8117625; doi:10.1186/s13041-021-00789-4)

STAI-S

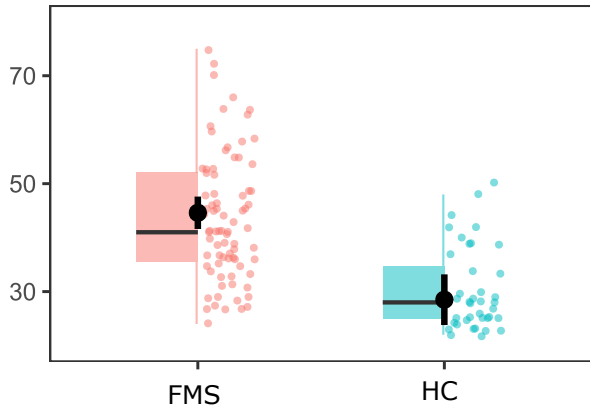

STAI-T

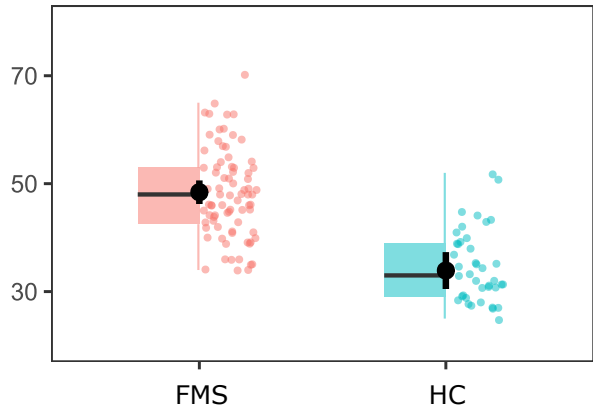

Supplement: Supplementary file 3 — Additional file 3: Fig. S1. Group differences in anxiety scores in fibromyalgia subjects (FMS) (n = 79) and healthy controls (HC) (n = 40). FMS showed significantly higher anxiety scores measured by the STAI (State-trait anxiety inventory) in both STAI-S (state subscale) and STAI-T (trait subscale). Plotted are black circles representing estimated marginal means with the associated black vertical lines being 95% confidence intervals. In the colored boxplots, the horizontal line represents the median, the upper and lower box representing the 25th (Q1) and 75th (Q3) percentile, i.e. the interquartile range (IQR), and whiskers represent Q1—and Q3 + 1.5*IQR. Raw data is plotted in the background. [file 13041_2021_789_MOESM3_ESM.pdf]

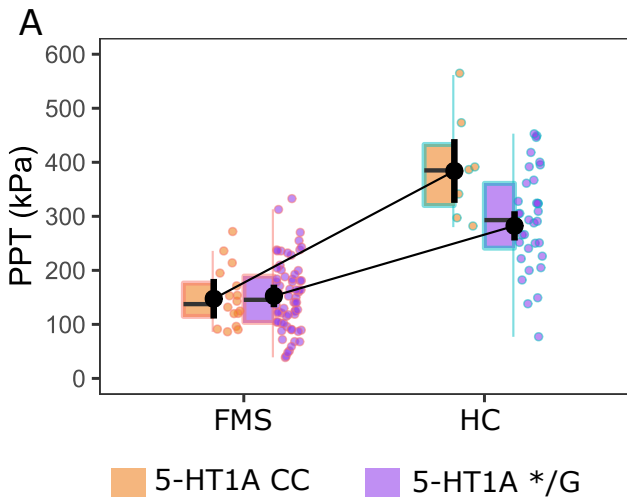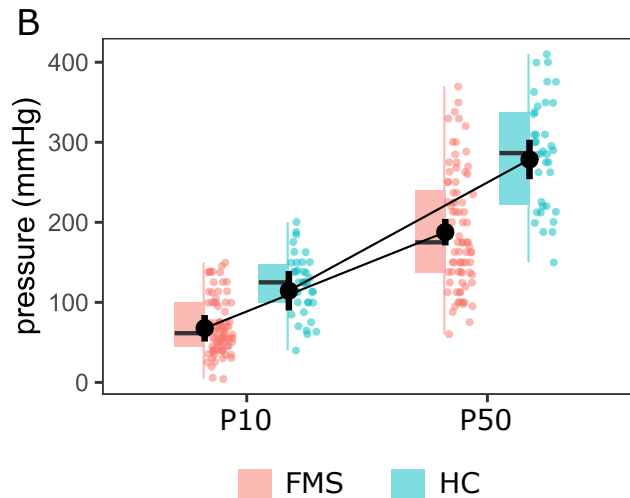

Supplement: Supplementary file 4 — Additional file 4: Fig. S2. Pain sensitivity measures in fibromyalgia subjects (FMS) (n = 80) and healthy controls (HC) (n = 40). A) In pressure pain thresholds (PPT) using a handheld algometer, a group-by-5‐HT1A interaction was observed, with 5‐HT1A CC in HC showing higher PPTs than G-carriers. This effect was not observed in FMS. B) Input pressure delivered via a rapid cuff inflation system necessary to achieve 10/100 on a visual analogue scale (VAS), P10, and 50/100 VAS, P50, differed between groups depending on pressure level. Plotted are black circles representing estimated marginal means with the associated black vertical lines being 95% confidence intervals. In the colored boxplots, the horizontal line represents the median, the upper and lower box representing the 25th (Q1) and 75th (Q3) percentile, i.e. the interquartile range (IQR), and whiskers represent Q1—and Q3 + 1.5*IQR. Raw data is plotted in the background. kPa = kilopascal, mmHg = millimeter of mercury [file 13041_2021_789_MOESM4_ESM.pdf]
